# Clinical characteristics associated with gastrointestinal bleeding in neonates with laboratory-confirmed norovirus gastroenteritis

**DOI:** 10.3389/fpubh.2026.1856399

**Published:** 2026-08-25

**Authors:** Linzhou Zhu, Hanghang Peng, Yuhan Huang, Hui Huang, Xueping Zhu, Yueping Shen

**Affiliations:** 1 Department of Epidemiology and Biostatistics, School of Public Health, Medical College of Soochow University, Suzhou, China; 2 Department of Neonatology, Children's Hospital of Soochow University, Suzhou, China; 3 Department of Clinical Laboratory, Children's Hospital of Soochow University, Suzhou, China

**Keywords:** gastroenteritis, gastrointestinal bleeding, ionized calcium, neonate, norovirus

## Abstract

**Introduction:**

Norovirus (NoV) is a major cause of acute gastroenteritis in children, but pathogen-confirmed data in neonates remain limited, particularly regarding clinical differences associated with gastrointestinal bleeding.

**Methods:**

We conducted a single-center retrospective observational study of neonates younger than 28 days who were hospitalized at the Children's Hospital of Soochow University between December 31, 2024 and December 31, 2025, had positive stool NoV antigen results, and underwent confirmatory nucleic acid testing. After excluding clinically identifiable alternative causes of gastrointestinal bleeding, patients were classified into hemorrhagic and non-hemorrhagic groups. Baseline characteristics, clinical manifestations, comorbidities, and laboratory findings obtained within 24 h after admission were compared between groups. Laboratory variables showing significant between-group differences were entered into a multivariable logistic regression model.

**Results:**

A total of 139 neonates were included, comprising 52 in the hemorrhagic group and 87 in the non-hemorrhagic group. Among the hemorrhagic cases, 49 had overt gastrointestinal bleeding and three had isolated fecal occult blood positivity. The hemorrhagic group had a higher frequency of diarrhea and a lower frequency of jaundice. Hemoglobin, total bilirubin, indirect bilirubin, aspartate aminotransferase, the aspartate aminotransferase/alanine aminotransferase ratio, pH-corrected venous ionized calcium, chloride, and lactate were lower in the hemorrhagic group, whereas blood base excess was higher. In multivariable logistic regression, each 0.1 mmol/L increase in ionized calcium was associated with lower odds of the hemorrhagic phenotype (OR = 0.624, 95% CI: 0.410–0.950; *P* = 0.028).

**Discussion:**

Neonates with laboratory-confirmed NoV gastroenteritis showed different clinical and laboratory characteristics according to the presence of gastrointestinal bleeding. The association with lower ionized calcium should be interpreted cautiously because of the retrospective design and potential residual confounding.

## 1 Introduction

Norovirus (NoV) is a leading cause of acute gastroenteritis worldwide and contributes substantially to pediatric morbidity and hospitalization, particularly among infants and young children (1–3). Following the widespread implementation of rotavirus vaccination, NoV has remained an important cause of pediatric gastroenteritis, although changes in its absolute prevalence have varied across settings (1, 2, 4). However, the clinical characteristics of laboratory-confirmed NoV infection in neonates remain insufficiently described.

Neonates represent a clinically distinct population in whom enteric infections may present with subtle or nonspecific manifestations. Immaturity of the intestinal epithelium and the developing mucosal immune system may contribute to variable or nonspecific clinical presentations (5, 6). Gastrointestinal bleeding is an important warning sign in neonatal practice because it requires differentiation from conditions such as necrotizing enterocolitis, cow's milk protein allergy, anal fissure, swallowed maternal blood, and coagulation disorders (7). Nevertheless, the clinical features associated with gastrointestinal bleeding among neonates with confirmed NoV gastroenteritis remain unclear.

Previous studies have mainly focused on older infants and children, epidemiological burden, molecular characteristics, or outbreak patterns (1–4). Evidence specific to neonatal NoV remains sparse; in a meta-analysis of viral infections and neonatal necrotizing enterocolitis (NEC), the NoV subgroup was based on only two studies (8). Few studies have directly compared neonates with and without gastrointestinal bleeding in terms of clinical manifestations and laboratory findings.

Therefore, this study aimed to compare the clinical and laboratory characteristics of neonates with laboratory-confirmed NoV gastroenteritis according to the presence or absence of gastrointestinal bleeding and to explore laboratory factors associated with the hemorrhagic phenotype.

## 2 Materials and methods

### 2.1 Study design and participants

This was a single-center retrospective observational study conducted at the Children's Hospital of Soochow University. Neonates younger than 28 days who were hospitalized between December 31, 2024 and December 31, 2025 and had clinically suspected gastroenteritis underwent stool NoV antigen testing, with positive results confirmed by NoV-specific nucleic acid testing (9).

Eligible patients were required to have laboratory-confirmed NoV infection together with compatible gastrointestinal manifestations. Neonates were excluded if they had confirmed or clinically suspected necrotizing enterocolitis, gastrointestinal bleeding attributable to cow's milk protein allergy, anal fissure, swallowed maternal blood, coagulation disorders, vitamin K deficiency bleeding, or another established cause. Patients with severe congenital anomalies, inherited metabolic disorders, other underlying diseases likely to affect gastrointestinal manifestations, or missing key grouping or clinical data were also excluded.

Patients were classified according to the presence or absence of gastrointestinal bleeding. The hemorrhagic group included neonates with overt gastrointestinal bleeding or isolated fecal occult blood positivity, whereas neonates without either finding were classified into the non-hemorrhagic group.

### 2.2 Definitions

NoV gastroenteritis was defined on the basis of pathogen confirmation and compatible gastrointestinal manifestations. Overt gastrointestinal bleeding was defined as clinically documented rectal bleeding or grossly bloody stools, whereas isolated fecal occult blood positivity referred to a positive fecal occult blood test without visible blood in the stool.

Alternative causes of gastrointestinal bleeding, including necrotizing enterocolitis, were assessed using feeding and clinical history, physical examination, abdominal radiography and ultrasonography, coagulation tests, vitamin K administration history, and the Apt test when indicated; modified Bell staging criteria were applied when necrotizing enterocolitis was suspected and sufficient clinical and imaging data were available (10, 11).

The diagnostic criteria for atrial septal defect, anemia, myocardial injury, urinary tract infection, metabolic acidosis, respiratory failure, and heart failure were based on standard neonatal practice and the reference text *Practical Neonatology* (12).

### 2.3 Data collection

Electronic medical records were reviewed retrospectively. The collected data included demographic and perinatal characteristics, such as sex, gestational age, birth weight, cesarean delivery, small-for-gestational-age status, admission weight, birth asphyxia, prolonged rupture of membranes greater than 18 h, and pre-admission feeding pattern; clinical manifestations at presentation, including vomiting, diarrhea, fever, and jaundice; comorbidities and complications during hospitalization, including pneumonia, anemia, myocardial injury, urinary tract infection, metabolic acidosis, respiratory failure, heart failure, and atrial septal defect; and laboratory indices obtained within 24 h of admission, including routine blood parameters, liver function indices, myocardial enzyme markers, blood gas parameters, electrolytes, coagulation parameters, and lactate (13).

Pre-admission feeding patterns were classified as exclusive breastfeeding, exclusive formula feeding, or mixed feeding. Exclusive breastfeeding included both direct breastfeeding and feeding with expressed breast milk. Additional microbiological investigations, including stool culture and testing for other pathogens, were performed according to clinical indications.

### 2.4 Statistical analysis

All statistical analyses were performed using SPSS version 26.0 (IBM Corp., Armonk, NY, USA). The normality of continuous variables was assessed using the Shapiro–Wilk test before between-group comparisons. Normally distributed variables are presented as mean ± standard deviation and were compared using the independent-samples *t*-test, whereas non-normally distributed variables are presented as median and interquartile range and were compared using the Mann–Whitney *U*-test. Categorical variables are presented as *n* (%) and were compared using the chi-square test or Fisher's exact test, as appropriate.

Laboratory variables with significant between-group differences in univariable analyses were entered into a multivariable logistic regression model to identify factors independently associated with the hemorrhagic phenotype. Odds ratios (ORs) and 95% confidence intervals (CIs) were calculated. All tests were two-sided, and *P* < 0.05 was considered statistically significant.

### 2.5 Ethics statement

This study was approved by the Ethics Committee of Children's Hospital of Soochow University (2022CS009). Written informed consent was obtained from the guardians of the neonates. All procedures were conducted in accordance with the Declaration of Helsinki and its later amendments or comparable ethical standards.

## 3 Results

### 3.1 Baseline characteristics

During the study period, 139 neonates with laboratory-confirmed NoV gastroenteritis were included. Among them, 52 were classified into the hemorrhagic group and 87 into the non-hemorrhagic group. Of the 52 neonates in the hemorrhagic group, 49 had overt gastrointestinal bleeding and three had isolated fecal occult blood positivity.

The baseline characteristics of the two groups are summarized in Table 1. There were no significant between-group differences in sex, gestational age, birth weight, cesarean delivery, small-for-gestational-age status, admission weight, birth asphyxia, prolonged rupture of membranes greater than 18 h, pre-admission feeding pattern, or atrial septal defect (all *P* > 0.05).

**Table 1:** Baseline characteristics of neonates in the hemorrhagic and non-hemorrhagic groups.

Variable	Hemorrhagic group (*n* = 52)	Non-hemorrhagic group (*n* = 87)	*P*-value
Male, *n* (%)	36 (69.2)	47 (54.0)	0.077
Gestational age (weeks), mean ± SD	38.01 ± 2.34	38.23 ± 1.82	0.536
Birth weight (g), mean ± SD	3,183.30 ± 605.54	3,187.79 ± 641.68	0.968
Cesarean section, *n* (%)	23 (44.2)	47 (54.0)	0.246
Small for gestational age, *n* (%)	5 (9.6)	7 (8.0)	0.766
Admission weight (kg), mean ± SD	3.63 ± 0.75	3.53 ± 0.73	0.429
Birth asphyxia, *n* (%)	3 (5.8)	3 (3.4)	0.791
Prolonged rupture of membranes (>18 h), *n* (%)	3 (5.8)	5 (5.7)	1.000
Pre-admission feeding pattern, *n* (%)	—	—	0.606
Exclusive breastfeeding	10 (19.2)	17 (19.5)	—
Exclusive formula feeding	23 (44.2)	45 (51.7)	—
Mixed feeding	19 (36.5)	25 (28.7)	—
Atrial septal defect, *n* (%)	19 (36.5)	26 (29.9)	0.417

Data are presented as mean ± standard deviation or *n* (%), as appropriate.

SD, standard deviation.

### 3.2 Clinical manifestations, comorbidities, and complications

Clinical manifestations and in-hospital comorbidities are shown in Table 2. Diarrhea was more frequent in the hemorrhagic group than in the non-hemorrhagic group (*P* = 0.021), whereas jaundice was less frequent in the hemorrhagic group (*P* = 0.004). There were no significant between-group differences in vomiting, fever, pneumonia, anemia, myocardial injury, urinary tract infection, metabolic acidosis, respiratory failure, or heart failure (all *P* > 0.05).

**Table 2:** Clinical features, comorbidities, and complications of neonates with norovirus gastroenteritis.

Variable	Hemorrhagic group (*n* = 52)	Non-hemorrhagic group (*n* = 87)	*P*-value
Vomiting	4 (7.7)	14 (16.1)	0.154
Diarrhea	32 (61.5)	36 (41.4)	0.021
Fever	4 (7.7)	16 (18.4)	0.136
Jaundice	25 (48.1)	63 (72.4)	0.004
Pneumonia	28 (53.8)	54 (62.1)	0.306
Anemia	17 (32.7)	21 (24.1)	0.274
Myocardial injury	14 (26.9)	20 (23.0)	0.602
Urinary tract infection	1 (1.9)	3 (3.4)	0.520
Metabolic acidosis	3 (5.8)	2 (2.3)	0.271
Respiratory failure	0 (0.0)	3 (3.4)	0.242
Heart failure	0 (0.0)	1 (1.1)	0.626

Data are presented as *n* (%).

### 3.3 Laboratory test results

Laboratory findings obtained within 24 h after admission are presented in Table 3. No significant between-group differences were observed in white blood cell count, platelet count, eosinophil percentage, absolute neutrophil count, absolute lymphocyte count, absolute monocyte count, total protein, albumin, direct bilirubin, alanine aminotransferase, lactate dehydrogenase, bile acids, creatine kinase-MB, high-sensitivity troponin T, myoglobin, pH, sodium, potassium, blood glucose, prothrombin time, activated partial thromboplastin time, or fibrinogen (all *P* > 0.05).

**Table 3:** Laboratory findings within 24 h of admission in neonates with norovirus gastroenteritis.

Variable	Hemorrhagic group (*n* = 52)	Non-hemorrhagic group (*n* = 87)	*P*-value
White blood cell count ( × 10^9^/L)	9.99 ± 3.10	11.12 ± 4.22	0.095
Hemoglobin (g/L)	133.62 ± 31.92	147.53 ± 35.22	0.021
Platelet count ( × 10^9^/L)	355.62 ± 133.81	328.80 ± 108.52	0.199
Eosinophil percentage (%)	3.74 ± 2.36	3.89 ± 2.67	0.744
Absolute neutrophil count ( × 10^9^/L)	4.09 ± 2.49	5.13 ± 3.63	0.072
Absolute lymphocyte count ( × 10^9^/L)	4.40 ± 1.65	4.03 ± 1.68	0.208
Absolute monocyte count ( × 10^9^/L)	1.17 ± 0.58	1.35 ± 0.69	0.105
Total protein (g/L)	52.19 ± 4.44	52.76 ± 4.91	0.499
Albumin (g/L)	37.12 ± 2.44	36.47 ± 3.27	0.219
Total bilirubin (μmol/L)	140.70 ± 115.66	196.93 ± 110.71	0.005
Indirect bilirubin (μmol/L)	125.99 ± 111.80	181.37 ± 109.59	0.005
Direct bilirubin (μmol/L)	14.71 ± 7.37	15.44 ± 9.57	0.635
Alanine aminotransferase (U/L)	17.51 ± 9.53	16.99 ± 15.09	0.823
Aspartate aminotransferase (U/L)	37.20 ± 10.95	46.56 ± 36.07	0.026
AST/ALT ratio	2.60 ± 1.47	3.62 ± 3.41	0.016
Lactate dehydrogenase (U/L)	434.61 ± 268.04	496.54 ± 229.10	0.153
Bile acids (μmol/L)	17.60 ± 40.69	14.78 ± 38.20	0.687
Creatine kinase-MB (U/L)	8.16 ± 3.83	9.53 ± 8.63	0.291
High-sensitivity troponin T (pg/mL)	78.70 ± 68.08	61.66 ± 43.48	0.116
Myoglobin (ng/mL)	37.74 ± 20.85	42.51 ± 41.63	0.586
pH	7.51 ± 0.05	7.50 ± 0.06	0.226
Base excess of blood (mmol/L)	−2.05 ± 2.34	−3.09 ± 2.57	0.019
Sodium (mmol/L)	135.23 ± 2.04	135.98 ± 2.77	0.095
Potassium (mmol/L)	4.44 ± 0.41	4.40 ± 0.44	0.590
pH-corrected venous ionized calcium (mmol/L)	1.05 ± 0.11	1.14 ± 0.19	0.001
Chloride (mmol/L)	107.14 ± 2.93	108.40 ± 2.78	0.013
Lactate (mmol/L)	3.29 ± 1.39	3.94 ± 1.44	0.010
Blood glucose (mmol/L)	4.62 ± 1.11	4.34 ± 1.25	0.184
Prothrombin time (s)	10.56 ± 4.93	9.82 ± 5.71	0.428
Activated partial thromboplastin time (s)	38.16 ± 9.30	40.71 ± 7.10	0.075
Fibrinogen (g/L)	2.59 ± 2.04	2.54 ± 0.88	0.830

Data are presented as mean ± standard deviation.

AST, aspartate aminotransferase; ALT, alanine aminotransferase.

Compared with the non-hemorrhagic group, the hemorrhagic group had lower hemoglobin (*P* = 0.021), total bilirubin (*P* = 0.005), indirect bilirubin (*P* = 0.005), aspartate aminotransferase (*P* = 0.026), AST/ALT ratio (*P* = 0.016), pH-corrected venous ionized calcium (*P* = 0.001), chloride (*P* = 0.013), and lactate levels (*P* = 0.010), together with higher blood base excess values (*P* = 0.019).

### 3.4 Multivariable logistic regression analysis

Laboratory indices showing significant between-group differences in univariable analyses were entered into a multivariable logistic regression model. Lower pH-corrected venous ionized calcium remained associated with the hemorrhagic phenotype. Each 0.1 mmol/L increase in ionized calcium was associated with lower odds of the hemorrhagic phenotype (β = −0.471, SE = 0.214, OR = 0.624, 95% CI: 0.410–0.950; *P* = 0.028), as detailed in Table 4.

**Table 4:** Multivariable analysis of laboratory variables between the two groups.

Variable	*P*-value	OR	95% CI
Total bilirubin	0.544	0.982	0.928–1.040
Indirect bilirubin	0.479	1.022	0.963–1.084
AST	0.207	1.015	0.992–1.038
AST/ALT ratio	0.883	1.015	0.828–1.245
Base excess of blood	0.777	0.970	0.785–1.198
Ionized calcium	0.028	0.624	0.410–0.950
Chloride	0.892	1.014	0.834–1.231
Lactate	0.111	1.292	0.943–1.770
Hemoglobin	0.619	0.996	0.980–1.012

OR, odds ratio; CI, confidence interval; AST, aspartate aminotransferase; ALT, alanine aminotransferase. The OR for ionized calcium represents each 0.1 mmol/L increase.

## 4 Discussion

In this single-center study of hospitalized neonates with laboratory-confirmed NoV gastroenteritis, hemorrhagic and non-hemorrhagic cases differed in both clinical presentation and laboratory profile. The hemorrhagic phenotype was characterized by a higher frequency of diarrhea, a lower frequency of jaundice, and several differences in hematologic, biochemical, electrolyte, and acid-base indices. Notably, lower pH-corrected venous ionized calcium remained associated with the hemorrhagic phenotype in the multivariable model.

These findings suggest that neonatal NoV gastroenteritis may present with clinically distinguishable patterns. Although NoV is an important cause of pediatric acute gastroenteritis, evidence specific to neonates remains limited (1–3, 8). Neonates differ from older children in intestinal epithelial and mucosal immune development (5, 6). In the present study, the terms hemorrhagic and non-hemorrhagic refer to clinical phenotypes based on gastrointestinal bleeding manifestations and do not represent distinct NoV genotypes, strains, or molecular subtypes.

The higher frequency of diarrhea in the hemorrhagic group may reflect more pronounced gastrointestinal involvement. NoV infection can disrupt epithelial integrity and alter intestinal absorption and secretion, while the immature neonatal intestine may be particularly susceptible to mucosal injury (5, 14). However, the present observational data cannot establish that NoV directly caused the bleeding manifestations, and the possible contributions of disease severity or concomitant infection should also be considered.

We also observed lower total bilirubin and indirect bilirubin levels in the hemorrhagic group, together with lower AST and AST/ALT ratio values. These findings suggest that neonates with hemorrhagic disease may exhibit a metabolic and hepatic profile distinct from that of non-hemorrhagic cases. In neonates, bilirubin metabolism is strongly influenced by hepatic immaturity and enterohepatic circulation (15). Although the clinical significance of these differences remains uncertain, they further support the possibility that the hemorrhagic phenotype represents a broader systemic response pattern rather than an isolated bleeding manifestation.

Hemoglobin was lower in the hemorrhagic group, which is clinically plausible given the presence of gastrointestinal bleeding. However, hemoglobin did not remain independently associated with the hemorrhagic phenotype after multivariable adjustment, suggesting that it may reflect a bleeding-associated consequence rather than an independent discriminative factor.

The most notable laboratory finding was the association between lower pH-corrected venous ionized calcium and the hemorrhagic phenotype. Ionized calcium is the biologically active circulating fraction; hypocalcemia is common in critical illness, and calcium signaling is central to vascular homeostasis (16, 17). Nevertheless, the present findings do not demonstrate that reduced ionized calcium causes gastrointestinal bleeding or contributes directly to its development. The observed association may be influenced by neonatal causes of hypocalcemia, including prematurity and nutritional or metabolic factors, as well as by sepsis or illness severity (18, 19). Ionized calcium is also sensitive to pH and pre-analytical conditions, including sample handling, anticoagulant exposure, and delays in analysis (13). In addition, the temporal relationship between the reduction in ionized calcium and the onset of bleeding could not be determined. Therefore, ionized calcium should be regarded as an associated laboratory finding rather than a confirmed causal or predictive biomarker.

Chloride, lactate, and blood base excess also differed between groups in univariable analyses. However, none remained independently associated with the hemorrhagic phenotype in the multivariable model, and their clinical relevance requires further investigation. Pre-admission feeding patterns did not differ significantly between the two groups. Exclusive breastfeeding did not preclude NoV infection in this cohort. NoV can be transmitted through direct contact with infected individuals and indirectly through contaminated hands, environmental surfaces, and other fomites (14). However, household contacts, breast milk, and environmental samples were not tested, and the source of infection could not be determined. In addition, a standardized multiplex gastrointestinal polymerase chain reaction (PCR) panel was not performed uniformly in all patients, and additional pathogen testing was guided by clinical indications. Therefore, undetected concomitant infections cannot be completely excluded.

This study provides a new perspective on the clinical phenotypes of neonatal NoV infection by identifying significant differences between hemorrhagic and non-hemorrhagic cases, including an association between lower pH-corrected venous ionized calcium and the hemorrhagic phenotype. However, there are several limitations to this study. First, it is a single-center, retrospective study with a relatively small sample size, and the results may be influenced by regional factors and selection biases. Second, the hemorrhagic group included both overt gastrointestinal bleeding and a small number of cases with isolated fecal occult blood positivity, which may represent different levels of clinical severity. Finally, several neonatal confounders, including hydration status, sepsis severity, nutritional status, and electrolyte supplementation, were not fully captured, while the lack of uniform multiplex pathogen testing and viral sequencing limited the assessment of concomitant infections and genotype-related differences. Multicenter prospective studies with standardized pathogen testing, clear temporal assessment, and viral genotyping are needed to validate these findings.

## Data Availability

The datasets generated and analyzed during the current study are not publicly available because they contain sensitive clinical information from neonatal patients. De-identified data may be made available from the corresponding authors upon reasonable request, subject to applicable institutional and ethical requirements.
